# RNA editing system: Balancing altruistic antiviral defense and fitness trade‐offs in fungi

**DOI:** 10.1002/mlf2.70057

**Published:** 2025-12-18

**Authors:** Yanfei Du, Daohong Jiang, Huiquan Liu

**Affiliations:** ^1^ State Key Laboratory for Crop Stress Resistance and High‐Efficiency Production, College of Plant Protection Northwest A&F University Yangling China; ^2^ State Key Laboratory of Agricultural Microbiology, Hubei Key Laboratory of Plant Pathology, Huazhong Agricultural University Hubei Hongshan Laboratory Wuhan China

**Keywords:** altruism, antiviral defense, fitness trade‐offs, OLD‐ZAO, RNA editing

RNA editing is a posttranscriptional process that alters genetic information encoded in genomic DNA. While most known RNA editing occurs in organellar RNAs, adenosine‐to‐inosine (A‐to‐I) editing is one of the few types that modify nuclear‐encoded mRNAs^1^, ^2^. Since inosine is interpreted as guanosine (G) during translation, A‐to‐I editing can recode proteins, adding a regulatory layer beyond the central dogma. This raises a key question: What evolutionary advantage does RNA editing provide? Current hypotheses suggest that RNA editing allows regulated production of multiple protein isoforms from a single gene, helping resolve fitness trade‐offs from pleiotropy or genetic conflict^3^, ^4^.

A‐to‐I mRNA editing is the most prevalent and well‐studied editing mechanism in animals, catalyzed by ADAR (adenosine deaminase acting on RNA) enzymes (Figure 1)^5^. The ancestral and conserved function of ADAR editing is to suppress aberrant antiviral immune responses triggered by transposable element (TE)‐derived double‐stranded RNAs (dsRNA)^6^. Interestingly, ADAR‐independent A‐to‐I mRNA editing has also been reported in fungi, with thousands to tens of thousands of editing sites identified per species, particularly during sexual reproduction^7^, ^8^. This process is critical for fungal sexual development^3^. A recent study has identified the Tad2‐Tad3‐Ame1 (tRNA‐specific adenosine deaminase 2/3 and activator of mRNA editing 1) ternary complex as the machinery responsible for A‐to‐I mRNA editing in Sordariomycete fungi^9^. The Tad2‐Tad3 complex is a conserved heterodimeric deaminase that typically edits the wobble position (A34) of tRNAs in eukaryotes. During sexual reproduction, the stage‐specific cofactor Ame1 facilitates the editing of mRNAs by the Tad2‐Tad3 complex through its interaction with the N‐terminal domain of Tad3 (Figure 1)^9^. Notably, both animals and fungi have independently evolved their A‐to‐I mRNA editing systems from ancestral tRNA‐specific adenosine deaminases^10^. It is hypothesized that the emergence of the animal ADAR editing system may have been driven by a development‐defense trade‐off associated with TE activation during the evolution of multicellularity^10^. Similarly, the emergence of the fungal Tad2‐Tad3‐Ame1 editing system may have been driven by a survival‐reproduction trade‐off posed by environmental changes during the End‐Permian warming^10^.

**Figure 1:**
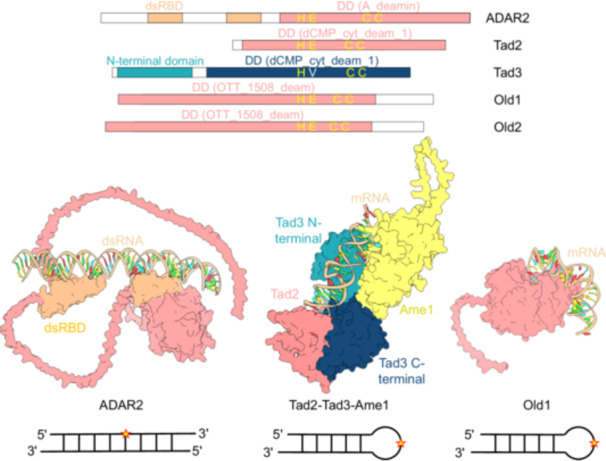
Domain architecture, structural models, and RNA substrate preferences of ADAR2, Tad2/3, and Old1/2 RNA editing systems. Upper panel: schematic domain organization of ADAR2, Tad2/3, and Old1/2. DD, deaminase domain (domain family annotated in the Pfam database); dsRBD, double‐stranded RNA‐binding domain. The Tad3 C‐terminal deaminase domain (dCMP_cyt_deam_1) is catalytically inactive (dark blue) due to a Glu (E)‐to‐Val (V) substitution in the conserved active‐site tetrad (His–Glu–Cys–Cys). Lower panels: AlphaFold3‐predicted tertiary structures of the three editing systems in complex with their preferred RNA substrates and their simplified secondary structures of the preferred RNA substrates. For each enzyme, the protein domains are shown in distinct colors, and the bound RNA is displayed to illustrate substrate preference. Ame1 is the activator of Tad2/3 mRNA editing. Representative simplified secondary structures of the preferred RNA substrates for each editing system are shown below each of the predicted tertiary structures. Horizontal lines represent the RNA sequence, with 5′ and 3′ indicating sequence direction, and vertical lines indicate base pairing. Stars denote edited adenosine positions.

## THE OLD‐ZAO MODULE AND PSC EDITING: ALLEVIATING FITNESS COSTS ASSOCIATED WITH ANTIVIRAL DEFENSE

**Figure 2:**
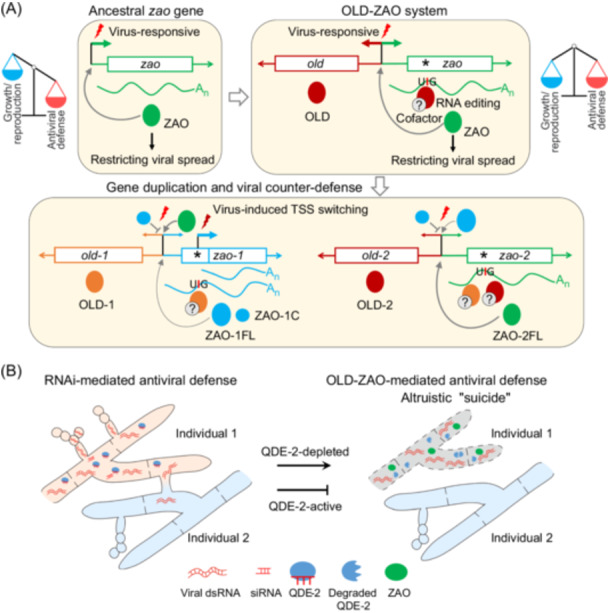
Evolutionary and functional model of OLD‐ZAO‐mediated altruistic antiviral defense in *Neurospora crassa*. (A) Evolutionary trajectory and viral counter‐defense targeting the OLD‐ZAO module. While the ancestral *zao* gene is responsive to viral infection, its leaky expression under virus‐free conditions reduces host fitness. The RNA‐editing enzyme OLD (which likely requires cofactors) introduces premature stop codons (PSCs, *) in *zao*, enabling PSC‐mediated repression under normal conditions. Upon viral infection, RNA editing corrects these PSCs, allowing full‐length ZAO expression. This OLD‐ZAO module enhances regulatory precision and minimizes fitness costs from constitutive ZAO activity. The OLD‐ZAO module has undergone gene duplication. One pair, *old‐1*_*zao‐1*, evolved rapidly in *N. crassa*, resulting in attenuated ZAO‐1 activity and relaxed OLD‐1 substrate specificity. As a counter‐defense, some mycoviruses induce transcriptional start site (TSS) switching in *zao‐1*, producing a truncated isoform (ZAO‐1C) that competitively inhibits full‐length ZAO‐1 and ZAO‐2 (ZAO‐1FL and ZAO‐2FL) by occupying their DNA‐binding sites, thereby suppressing antiviral responses. (B) Altruistic suicide by OLD‐ZAO as an antiviral backup upon RNAi suppression. Unlike RNAi, which cell‐autonomously restricts viral replication, OLD‐ZAO activation triggers severe growth and reproductive defects—an altruistic “suicidal” strategy that limits viral spread at the population level. Due to its detrimental effects on the individual, this system is tightly regulated: the core RNAi factor QDE‐2 (quelling defective 2, Argonaute) suppresses OLD‐ZAO activity under normal conditions, and only strong viral infection—which triggers QDE‐2 degradation—fully activates the defense.

In a recent study, a third mechanistically distinct A‐to‐I editing system, mediating antiviral defense in *Neurospora crassa*, was reported (Figure 2A)^11^. This system features two conserved OLD‐ZAO gene pairs (*old‐1_zao‐1* and *old‐2_zao‐2*). The OLD (OTT_1508‐like deaminase) proteins contain an OTT_1508‐like deaminase domain with a catalytic center characterized by the HXEXnCXXC motif, which is conserved in both Tad2 and ADARs^12^. Unlike ADARs, which preferentially act on dsRNA substrates, OLD proteins show an mRNA substrate preference similar to the Tad2–Tad3–Ame1 complex, favoring RNA hairpin loops and a uridine at the −1 position (Figure 1)^8^, ^11^. However, in contrast to the genome‐wide editing mediated by Tad2–Tad3–Ame1^8^, OLD proteins induce only a limited number of editing events, primarily targeting their cognate ZAO (zinc fingers adjacent to Old) transcription factors to orchestrate antiviral responses^11^. The system operates through precise premature‐stop codon correction (PSC) editing, converting UAG to UIG in *zao‐1/2* transcripts to enable full‐length protein (ZAO‐1FL and ZAO‐2FL) expression^11^. While OLD‐2 exclusively edits *zao‐2*, OLD‐1 exhibits broader substrate specificity, targeting both *zao* loci and other genomic sites^11^. The need for unidentified cofactors for full activity suggests extra regulatory control.

The OLD‐ZAO system in fungi is an intricate antiviral defense mechanism that becomes transcriptionally active during infections by mycoviruses like *Neurospora crassa* fusarivirus 1 (NcFV1) and *Neurospora crassa* partitivirus 1 (NcPV1)^11^. This system is negatively regulated by Argonaute protein QDE‐2 (quelling defective‐2), a core component of classical RNA interference (RNAi)‐based antiviral defense^11^. In wild‐type *Neurospora*, these viruses usually cause asymptomatic infections. However, when *QDE‐2* is deleted, the high‐level expression of ZAO‐1FL and ZAO‐2FL from PSC‐edited transcripts leads to growth and reproductive defects^11^. ZAO‐2FL, and likely ZAO‐1FL, bind to the OLD‐ZAO genomic loci, inducing their expression and creating a positive feedback loop. Additionally, the *zao‐1* gene can produce shorter transcripts (*zao‐1C*) through alternative transcription start sites (TSSs), resulting in a truncated protein (ZAO‐1C) that retains zinc‐finger DNA‐binding domains but lacks the N‐terminal regulatory region^11^. ZAO‐1C may competitively inhibit ZAO‐1FL and ZAO‐2FL by occupying their DNA‐binding sites. Furthermore, mutual inhibition between different TSSs might suppress *zao‐1FL* transcription in favor of *zao‐1C*. The evolutionary conservation of the OLD‐ZAO module across diverse fungal lineages suggests that it plays a significant antiviral role^11^. The complexity of this system, involving both RNA editing and TSS switching, raises intriguing evolutionary questions.

Honda et al. demonstrated that the overexpression of edited versions of *zao‐1* (*zao‐1* ^*TGG*^) or *zao‐2* (*zao‐2* ^*TGG*^) results in severe growth and reproductive defects even in the absence of viral infection^11^. Similarly, Zou et al. found that the edited strain (*CHE1* ^*TGG*^) of the *CHE1* gene, a homolog of *zao* in *Fusarium graminearum*, exhibited severe growth and reproductive defects^13^. In addition to these experimental findings, Honda et al. further showed through phylogenetic analyses that the OLD‐ZAO system is present at least in *Eurotiomycetes*, *Sordariomycetes*, and one Basidiomycete species^11^, indicating that this system is evolutionarily conserved across multiple fungal lineages. Together, these findings highlight the conserved functional importance of the OLD‐ZAO system and raise the question of why PSC editing is favored over directly encoding the full‐length protein.

Given the significant adverse effects that *zao‐1* and *zao‐2* exert on fungal growth and reproduction, their expression must be tightly regulated. Although *zao* genes are induced by viral infection, virus‐responsive promoters alone cannot entirely prevent their expression under virus‐free conditions^11^, ^13^, potentially resulting in fitness reduction due to leaky expression. Studies in *F. graminearum* revealed that full‐length expression of certain genes is essential for sexual reproduction, yet low‐level expression during vegetative growth can cause defects under stress. Sexual stage‐specific A‐to‐I editing generates PSC codons in these genes, facilitating PSC editing and resolving survival‐reproduction trade‐offs^4^. A similar principle may apply to the evolution of the OLD‐ZAO system. Since *zao* is a functional gene for antiviral defense, the emergence of a premature UAG stop codon without concurrent PSC editing would likely impose a fitness cost and be eliminated by natural selection. Therefore, the most plausible scenario is that the pre‐existing A‐to‐I editing activity of OLD proteins could have relaxed the functional constraints on *zao* genes, thereby permitting the fixation of G‐to‐A mutations that created new editable UAG stop codons (PSC editing sites) (Figure 2A). Upon viral infection, virus‐responsive OLD expression edits the PSC codon in *zao* transcripts, ensuring full‐length ZAO proteins (ZAO‐1FL and ZAO‐2FL) are produced only when necessary. Thus, virus‐responsive PSC editing in *zao* genes, together with the unique OLD‐ZAO genomic arrangement, likely enhances precise cis‐regulatory control and mitigates fitness costs from unintended expression. Moreover, this evolutionary process highlights the close interplay between A‐to‐I editing and G‐to‐A mutations, as the formation of editable A sites depends on the presence of RNA editing. Furthermore, PSC editing mitigates the fitness cost caused by antiviral defense, allowing such mutations to be fixed by positive selection and maintained as part of the regulatory mechanism controlling ZAO expression.

Restorative RNA editing typically corrects harmful genomic mutations at the RNA level. According to neutral evolutionary theory, RNA editing activity may relax functional constraints on DNA, allowing mutations to accumulate and leading to restorative editing^14^. From this view, restorative RNA editing is considered nonadaptive, offering no fitness advantage and adding unnecessary complexity^14^. However, PSC editing of *zao* genes represents a novel example—alongside PSC editing of genes essential for sexual reproduction^4^—showing that restorative editing can confer adaptive advantages by resolving fitness trade‐offs.

## TSS SWITCHING: A SOPHISTICATED VIRAL COUNTER‐DEFENSE MECHANISM

The two conserved gene pairs of the OLD‐ZAO module in *Neurospora* likely originated from gene duplication (Figure 2A). This is supported by similarities in PSC editing site positions and functional similarities between ZAO‐1FL and ZAO‐2FL, and between OLD‐1 and OLD‐2^11^. After duplication, the *old‐1_zao‐1* module appears to have evolved rapidly, possibly due to relaxed selective pressure. Compared to OLD‐2, OLD‐1 affects a broader range of RNA editing targets^11^, reflecting decreased substrate specificity. Similarly, ZAO‐1 shows reduced transcription factor activity relative to ZAO‐2^11^. A notable distinction is that *zao‐1* uniquely expresses a shorter transcript termed *zao‐1C* via TSS switching (Figure 2A)^11^. This encodes a truncated protein, ZAO‐1C, functioning independently of RNA editing and negatively regulating antiviral responses. Honda et al. hypothesized that ZAO‐1C expression represents a symptom mitigation strategy facilitating asymptomatic viral infection^11^. This raises the evolutionary question: is ZAO‐1C a viral counter‐defense or a host adaptation to modulate antiviral responses?

NcFV1 infection is asymptomatic in wild‐type *Neurospora* but becomes symptomatic in *∆qde‐2*, while NcPV1 infection remains asymptomatic in both^11^. Deletion of *zao‐1* results in symptomatic infections by both viruses^11^. QDE‐2 is degraded upon NcFV1 infection but remains stable during NcPV1 infection^11^, ^15^. Since QDE‐2 negatively regulates OLD‐ZAO‐mediated responses, if ZAO‐1C regulates antiviral responses, one would expect a negative correlation between ZAO‐1C expression and QDE‐2 abundance. However, *zao‐1* expression does not differ between wild type and *∆qde‐2* upon viral infection. Compared to NcFV1, NcPV1 induces lower *old‐1/2* expression but higher *zao‐1* expression in *∆qde‐2* ^11^. Since *zao‐1C* is the main *zao‐1* transcript, elevated *zao‐1* likely reflects increased ZAO‐1C. These results indicate that ZAO‐1C expression correlates with viral symptomatology, not QDE‐2 degradation. Thus, the TSS‐driven switch producing ZAO‐1C likely represents a viral counter‐defense employed by NcFV1 and NcPV1 to subvert host defenses, rather than a precise host adaptation. Viral infection may alter components of the core transcriptional machinery, transcription factor activity, epigenetic modifications, or chromatin accessibility, thereby inducing alternative TSSs utilization in *zao‐1*. Although the exact molecular pathways underlying this process remain unclear, they constitute an important direction for future research. A virus capable of degrading QDE‐2 but unable to induce ZAO‐1C would likely trigger robust antiviral responses. Compared to NcFV1, NcPV1 induces higher ZAO‐1C^11^, consistent with higher viral titers, yet remains asymptomatic. The discovery of the OLD‐ZAO antiviral system thus provides insights into both host defense and viral counter‐defense strategies.

## POPULATION‐LEVEL PROTECTION BY THE OLD‐ZAO SYSTEM: A GENETIC MODEL FOR ALTRUISM

Honda et al. showed that the OLD‐ZAO system is transcriptionally activated by mycoviruses, such as NcFV1 and NcPV1. Deletion of *qde‐2* strongly activates this system^11^, and NcFV1 infection induces QDE‐2 degradation^15^, suggesting that the virus evades RNAi by targeting QDE‐2 for destruction. Although the molecular details of virus‐induced OLD‐ZAO activation and QDE‐2 repression remain unclear, the regulatory logic is straightforward. Unlike RNAi, which restricts viral replication in a cell‐autonomous manner, activation of OLD‐ZAO causes severe growth and reproductive defects; thus, it must serve as a tightly regulated, fail‐safe antiviral mechanism. Under normal conditions, QDE‐2 represses *old‐zao* transcription, but during severe viral infection—when QDE‐2 is degraded—the OLD‐ZAO pathway is fully activated to counter infection (Figure 2B).

Activation of the OLD‐ZAO system causes severe defects, yet fails to suppress viral replication, as shown by elevated NcFV1 titers in *∆qde‐2* ^11^. Given that mycoviruses depend on both horizontal (via hyphal anastomosis) and vertical (through spores) transmission^16^, the OLD‐ZAO‐mediated response likely represents an altruistic “suicide” strategy, sacrificing infected individuals to limit viral spread at the population level (Figure 2B). From an evolutionary perspective, such altruistic traits can be maintained if they preferentially benefit genetically related individuals, in line with Hamilton's rule^17^. In fungi possessing the OLD‐ZAO system, such altruistic self‐sacrifice could operate through an unknown mechanism of “kind recognition”^18^, directing benefits toward individuals with the same genetic background. By sacrificing infected individuals, this system provides a population‐level evolutionary advantage. This population‐level advantage likely explains QDE‐2‐mediated suppression and the evolutionary conservation of the OLD‐ZAO system. The OLD‐ZAO system exemplifies biological altruism—a phenomenon challenging traditional Darwinian selection^19^. As such, it offers a valuable model for studying the genetic and evolutionary foundations of altruism in microbes.

In summary, the discovery of the antiviral‐responsive RNA editing system is a significant advance in eukaryotic biology. It reveals new dimensions of RNA editing diversity and fungal antiviral strategies, and provides compelling evidence for the adaptive significance of RNA editing in host‐virus coevolution. This discovery establishes a novel model for investigating both the molecular mechanisms of RNA editing and the evolutionary dynamics of altruistic defense.
